# Do Consumers Want Seaweed in Their Food? A Study Evaluating Emotional Responses to Foods Containing Seaweed

**DOI:** 10.3390/foods10112737

**Published:** 2021-11-09

**Authors:** Rachael Moss, Matthew B. McSweeney

**Affiliations:** School of Nutrition and Dietetics, Acadia University, Wolfville, NS B4P 2K5, Canada; 145961m@acadiau.ca

**Keywords:** food product development, functional foods, check all that apply, emotions, consumer perceptions

## Abstract

Seaweeds are nutrient-dense marine organisms that have been proposed as a key ingredient to produce new functional foods. This study’s first objective was to identify consumers’ emotional responses and purchase intent towards a variety of food products containing seaweed. The secondary objective was to evaluate how hunger status and lifestyle affect consumers’ emotional responses. Participants (n = 108) were asked to evaluate pictures of different food items containing seaweed (beef burger, cheddar cheese, fettuccine, fish filet, sausage, bread, yogurt, and dried seaweed) using the CATA variant of EsSense25 Profile^®^ and a purchase-intent scale. The consumers also answered questions about their hunger status, food neophobia, food-related lifestyle, as well as open-ended comment questions about seaweed. Participants’ purchase-intent scores were highest for bread and dried seaweed, which they associated with positive emotions. The participants disliked yogurt and sausage, indicating that they were disgusted with them. Participants believed seaweed could be added to fish, savoury, and cereal grains-based foods. The participants’ hunger status as well as their food neophobia and lifestyle impacted their emotional responses. Future research should continue to investigate how emotions affect purchase intent, how participant’s hunger status affects their emotions, and how participants’ lifestyle changes how they perceive new food products.

## 1. Introduction

Seaweeds, more formally known as macroalgae, are large colonies of diversified algae that grow in both freshwater and marine ecosystems around the world. Seaweeds have been on earth dating back to 1.2 billion years ago [1]. Macroalgae differ from microalgae, such as cyanobacteria and phytoplankton, in that they are large enough to be observable by the naked eye, ranging from a few millimetres to upwards of 30–50 m [2,3]. Seaweeds range in size, shape, and colour; they are mainly differentiated based on the ratio of colour pigments used for photosynthesis, which separates the various species of seaweed into green (Chlorophyta), red (Rhodophyta), and brown (Phaeophyta) varieties [4]. 

Seaweeds are nutrient-dense marine organisms [5] that are low in fat (by dry weight 0.51–2.8%) [6], and their fatty acid profiles are comprised of high levels of monounsaturated and polyunsaturated fatty acids [7,8]. Seaweeds are also excellent sources of protein [9,10,11,12]. Moreover, seaweeds are approximately 50% carbohydrates by dry weight [9,13]. Kumar et al. [6] found that in five species of brown macroalgae examined, the carbohydrate concentration was between 19.4–34.9% by dry weight. Of the carbohydrates present in seaweed, portions exist as insoluble and soluble fibre in unusually abundant amounts ranging from 29.3 to 50% dry weight [14,15]. Such high values are consistent with or exceed many commonly consumed fruits and vegetables. Seaweeds are also excellent sources of both macro- and micro-minerals, even more so than most terrestrial plants [14]. The most abundant vitamins in seaweed were found to be vitamin C, vitamin E, and vitamin A [13,16]. The most prominent macro-minerals found in seaweeds include sodium, calcium, and potassium [14,16]. The micro-minerals with the highest concentrations found in seaweed include iron, nickel, cobalt, copper, and manganese [6,16].

Based on their nutritional properties and sustainability, seaweeds have more frequently been incorporated into a variety of different food products [17,18,19,20]. Therefore, this study aimed to evaluate consumers’ emotional responses to foods containing seaweed. Interest in measuring product-related emotions has grown in recent years [21]. Past studies have found that consumers choice of food has an emotional component [22,23], and consumers’ emotions have been evaluated using the EsSense Profile^®^ [24]. The EsSense Profile^®^ asks participants to look at 39 common emotional terms and to score the intensity of each term. Jaeger et al. [25] built on this research and created a check-all-that-apply (CATA) variant of the EsSense Profile^®^. The CATA variant can overcome some of the limitations of the EsSense Profile^®^, including some consumers not experiencing certain words and rating them as “slight” or “moderate”, which will not add much product insight [26,27]. The CATA variant also requires less cognitive effort from the participants [25]. Additionally, the EsSense Profile^®^ was shortened to 25 words by Nestrud et al. [27] and called EsSense25. The EsSense25 method has been found to show good repeatability and replicability as well as being easy for the participants to understand [27]. Based on these results, this study used the CATA variant of the EsSense25 version.

A secondary objective was to evaluate how hunger and lifestyle affect the emotional responses of the participants. Humans tend to be aroused, alert, and irritable when hungry [28]. After eating a meal, humans are typically calm, lethargic, and their mood is more likely to be positive [29]. Emotions have been linked to the energy content of foods, and as the energy content of the food increased, negative emotions were more frequent [30]. This study aims to build on this work by asking participants to self-identify their hunger status and evaluate their emotional responses to different foods containing seaweed. In addition, lifestyle is a popular base for segmentation in consumer marketing, and as such, a food-related lifestyle (FRL) instrument was developed in the mid-1990s [31]. The FRL hopes to identify differences in how consumers view food and beverages. The FRL was originally a 69-item questionnaire [31]; however, it was shortened to 15 items [32] and was found to be cross-culturally valid. This study used the 15-item version of the FRL to evaluate whether different consumers (based on their lifestyle) use different emotions to describe the food products made with seaweed. 

The aim of this study was to evaluate consumers’ emotional responses to different food products containing seaweeds. A secondary objective was to evaluate how hunger status and lifestyle affects consumers’ emotional responses. Firstly, consumers identified how hungry they were and then evaluated their emotional responses to food products. The food products included were identified by a literature review of studies investigating the addition of seaweeds to food products. Consumers also identified which foods they thought should include seaweed as well as the sensory properties they associate with seaweeds using open-ended comment questions. Lastly, participants completed a food neophobia questionnaire [33], food-related lifestyle questionnaire [32], and demographic questions.

## 2. Materials and Methods

### 2.1. Food Products

A literature review was completed to identify which food items should be included in the survey. The literature review was focused on studies that had included sensory analysis trials and products containing seaweed. The keywords used were “seaweed sensory analysis”, “seaweed sensory evaluation”, and “seaweed consumer perception.” The search was refined by year (from 2000 to present) and revealed 19 studies (Supplementary Table S1). The studies are identified in Supplementary Table S1, and the sensory method (either consumer panel, semi-trained panel, or trained panel) is also provided. Based on the literature review, beef burger, cheddar cheese, fettuccine (noodles), fish filet, sausage, bread, and yogurt were selected and included in the questionnaire. Furthermore, dried seaweed without addition to any food products was included. 

### 2.2. Participants

Participants were recruited from the Annapolis Valley, Nova Scotia community using posted advertisements on social media sites. All participants (n = 108; Supplementary Table S2) were screened to ensure they did not work in a sensitive industry (food or sensory analysis). 

### 2.3. Questionnaire

Approval for the study was received from the Acadia University Research Ethics Board (REB 13-72). The participants first completed an informed consent form and then identified their hunger status using scales adapted from Friedman et al. [34]. Participants were then presented with a picture of each food product described above and a statement indicating that the product contained seaweed. For instance, above the picture of cheddar cheese, was the following statement: “Cheddar cheese made with added seaweed.” The participants completed a CATA question containing the 25 emotional terms in the EsSense25 Profile^®^ [27]. The question was worded as “How do you expect to feel after eating this product? Check or click all that apply” [25]. Participants were also asked their purchase intent for each sample ranging from 1 = “Definitely will not buy” to 9 = “Definitely will buy.” Then, the participants had the option to describe what they liked and disliked about the samples in an open-ended comment question. Participants were also asked to identify which foods they would add seaweed to, the flavours they associate with seaweed and seaweed-containing products, and the nutritional benefits of seaweed through open-ended comment questions. Additionally, participants completed a food neophobia scale [33], the 15-item FRL [32], and demographic questions. The questionnaire was presented using Compusense Cloud software (Guelph, Ontario, Canada).

### 2.4. Statistical Analysis

A two-way random factor (samples and participants) ANOVA followed by a Tukey’s HSD test was completed to determine any significant differences in liking among the different samples. The frequency of use of each emotional term used by the participants to describe each sample was calculated. Cochran’s Q test was used to identify significant differences among the samples for each of the emotional terms included in the CATA question. If there was a significant difference among the attributes, then post-hoc multiple pairwise comparisons were conducted using McNemar’s test with Bonferroni alpha adjustment. Correspondence analysis was performed on the total frequency count of the emotions for each product to identify relationships between the emotions and the samples [35]. A penalty lift analysis was carried out following the procedure by Meyners and Casture [36]; however, it was adapted by using the purchase intent.

The participant’s responses to the open-ended comment questions were visualized using the comment visualization tools (Word Cloud and Text Network) in the Compusense Cloud software. Additionally, the procedure by Fonseca et al. [37] was followed to refine the definitions. The procedure included (a) verifying spelling and correcting grammatical mistakes; (b) removing connectors and auxiliary terms; (c) reducing derivatives of the same term; and (d) grouping synonyms together. Lastly, recurring themes and key concepts in the responses were identified. Results were also discussed among the authors to reach a consensus.

The FNS was used to segment the participants based on their degree of food neophobia. The sum score of the ten questions from the FNS was calculated and participants were divided at the median score into either a high food neophobia (HFN) group (n = 54, sum score = 30–48) or a low food neophobia (LFN) group (n = 54, sum score = 12–29). The same procedure was followed to segment the population based on the FRL into higher food-related lifestyle (HFRL, n = 54, sum score = 82–101) and lower food-related lifestyle (LFRL, n = 54, sum score = 57–81). Lastly, the procedure was followed to divide the population based on the hunger scales into those who were high hunger (HH, n = 54, sum score = 15.9–29.7) and low hunger (LH, n = 54, sum score = 6.0–15.3). This method of population segmentation was modelled after Laaksonen et al. [38], who separated their participants into three approximately evenly sized groups based on sum scores. However, due to the number of participants (n = 108), the procedure by Romaniw et al. [39] was followed, and the participants were segmented at the median to achieve two groups based on each scale (FNS, FRL, and Hunger). To determine any significant differences in liking between the participant segments, *t*-tests (*p* = 0.05) were performed. Furthermore, statistical analysis of the responses to the CATA question from each participant segment (based on FNS, FRL, and Hunger) was performed as described above. All analyses were completed using XLSTAT software (Version 2021.2, New York, NY, USA) in Microsoft Excel^TM^.

## 3. Results and Discussion

### 3.1. Seaweed Product Development

The mean purchase-intent scores are listed in Table 1. The overall study population (n = 108) identified that they would be significantly more likely to purchase dried seaweed and bread made with seaweed over the other food items (*p* < 0.05). Additionally, the participants identified that they would prefer seaweed added to fish filets, cheese, and beef burgers rather than yogurt and sausages (*p* < 0.05). Interestingly, the dried seaweed by itself had a significantly higher purchase intent than all the food items except for bread. This finding disagrees with many statements from the studies in Supplementary Table S1, as the studies stated that seaweed should be incorporated into food items to increase the consumption of seaweed. These studies also discussed how incorporating seaweed into food items can increase the nutritional benefits of food products [40,41]. Based on the results of this study, consumers are more interested in seaweed on its own. Therefore, companies may want to look at increasing education about the sustainability of consuming dried seaweed instead of incorporating seaweed into food items [42,43]. However, the results may have differed if the participants’ consumed the products, as seaweed has been found to not be well liked by consumers [2]. Overall, most mean purchase-intent scores were quite low on the 9-point purchase intent scale, ranging from 4 to 6. This may indicate that the participants are not interested in purchasing seaweed-incorporated food items. Based on the purchase-intent scores, the bread and dried seaweed seem to be most likely to be purchased by the participants. Additionally, the seaweed-incorporated food items were not compared to conventional food items (without seaweed addition), and this would have indicated how the consumers’ purchase intent changed based on the seaweed addition. 

**Figure 1:**
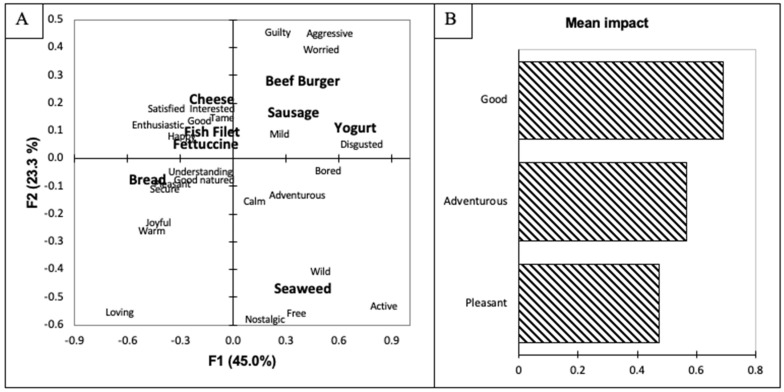
(**A**) Biplot representation of the seaweed-containing food items and emotional terms on the first two dimensions of the correspondence analysis for the overall population. (**B**) Penalty lift analysis of the emotional terms and willingness to pay based on the overall population’s evaluation of the seaweed-containing food items.

The emotional terms included in the CATA question were evaluated using correspondence analysis (CA), as found in Figure 1A. The biplot explained 68.3% of the variance, with the first dimension accounting for 45.0% and the second dimension for 23.3%. The first dimension separated the beef burger, sausage, and yogurt seaweed from the other food items (cheese, fish filet, fettuccine, and bread). The second dimension separated bread and dried seaweed from the other food items. The dried seaweed was associated with nostalgic, free, wild, active, and adventurous. In the open-ended comment question about the food items, participants stated they remembered their parents eating dried seaweed or dulse, which may explain why the dried seaweed was associated with nostalgia. Nostalgia has been found to be associated with pleasure and pleasantness [44], and this may explain why the purchase-intent scores for dried seaweed were significantly higher than all other food items except for bread (*p* < 0.05). Furthermore, participants identified that they had consumed dried seaweed before or were familiar with the product. Familiarity has been shown to increase consumers’ purchase intent [45]. Although familiarity was not explicitly asked about in the questionnaire, the results of the open-ended comment question indicate that participants are familiar with dried seaweed, which could have influenced the purchase-intent scores. In addition, Nova Scotia, Canada is located on the Atlantic Ocean, and many residents would be familiar with seaweed. Dried seaweed was also associated with adventurous, wild, active, and free. This result may have occurred as many of the participants stated that dried seaweed is a “great on-the-go snack” and also a “healthy snack that is part of an active lifestyle, like hiking.” 

The bread, which was the only food item not to be significantly different from the dried seaweed, was also on the negative side of the second dimension. The bread was associated with positive emotions, including understanding, good-natured, pleasant, secure, joyful, warm, and good. Similar to past studies, products associated with positive emotions were also associated with increased purchase-intent scores [46]. Bread had the highest purchase intent score (6.6 ± 1.2; Table 1) of all food products. Bread is also a very common product that consumers are familiar with, and as stated above, familiarity has been shown to increase consumers’ purchase intent [45]. The cheese, fish filet, and fettuccine food products were grouped together and associated with positive emotions, including satisfied, interested, tame, happy, and enthusiastic. In the open-ended comment questions, participants said the added seaweed would “match with the saltiness” of the cheese. The participants also stated that the seaweed would pair well with the fish filet, as “they are both from the ocean.” Additionally, participants believed that the seaweed would add nutritional benefits to the fettuccine and fish filets. Lastly, the beef burger, sausage, and yogurt were grouped and associated with negative emotions, including bored, disgusted, worried, aggressive, and guilty. This result was reinforced by the open-ended comment questions, in which participants expressed disgust with the seaweed being added to yogurt, sausages, and beef burgers. Moreover, participants’ disliking was found in the purchase-intent scores as well, as the yogurt and sausages scored significantly less than all of the other food items (*p* < 0.05). This result agrees with past studies that have found that negative emotions decrease consumers’ purchase intent [47,48,49]. Interestingly, although the beef burger was associated with negative emotions, the participants rated their purchase intent of beef burgers similar to the fettuccine, cheese, and fish filets.

A penalty lift analysis was conducted to determine if the participant’s emotions affected their willingness to purchase the different food items. The results of the penalty lift analysis can be seen in Figure 1B. The emotional terms good, adventurous, and pleasant led to an increase in the participants’ purchase-intent scores. The aforementioned emotions are identified as positive emotions [27] and indicate that positive emotions led to increased purchase intent. Furthermore, positive emotions are correlated with overall liking [21,50]. Past studies have found that pleasure significantly affects consumers’ satisfaction with a product [51,52], and although this study was investigating proposed food items, the same result was found as consumers’ purchase intent increased when they perceived positive emotions. 

Participants were also asked three open-ended comment questions, including what food product they would add seaweed to, what is the flavour of seaweed, and what nutritional benefits do seaweeds possess. The researchers then identified general categories based on the participants’ responses, which are provided in Table 2. The participants’ responses to which food products they would add seaweed to were separated into five categories, including fish, savoury, hide it, cereal grains, and seasoning. Firstly, many of the participants mentioned that seaweed should be incorporated into fish products as seaweed already tastes fishy. Many participants mentioned that seaweed is already used in sushi and therefore contributes well to fish products and dishes. This was an expected result, as many other studies have found that consumers associate seaweed with sushi [42,43,53]. Many participants also mentioned that seaweed would go well with savoury dishes, and some participants suggested that seaweed should be included in sausages. However, the purchase-intent scores for the sausages were the lowest of all food items (Table 1). The savoury category did have some responses that overlapped with the hide it category (Table 1), as some participants felt that savoury dishes would work well with the flavour of the seaweed, while others thought savoury dishes would mask the flavour of the seaweed. Past studies have suggested that seaweed could be used as a source of umami flavour in dishes [54,55], and this agrees with participants’ responses. 

Other participants identified that seaweed should be added to products where the flavour of seaweed could be masked by the foods’ sensory properties (hide it category; Table 2). The food most often cited that could mask the taste of the seaweed was pasta sauce. This suggested food item may be due to its association with pasta and with fettuccine being included in the questionnaire, therefore prompting the association. This result also agrees with past research that suggested that seaweed should be added to products for its nutritional benefits and for its flavour to be masked or minimized [56,57,58]. As identified by Rioux et al. [56], in most cases, seaweed is added to develop new functional foods or increase the sustainability of the food item rather than to enhance the sensory properties. However, the addition of seaweed to pasta also reduces the cooking loss [57]. The participants also identified that seaweed could be added to other cereal grain-based products. These responses by the participants also agree with past studies investigating seaweed addition to noodles [58], bread [59], extruded products [60], and muffins [61]. However, the addition of seaweed to bread and muffins has also been shown to negatively impact consumer acceptability [59,61]. Lastly, participants identified that seaweed could be used as a seasoning on a variety of different products to enhance the flavour of the dish or to add a salty taste. In addition, some participants discussed how seaweed could add texture to a dish or a “crunch factor.” The responses to this question mainly discussed the taste, aroma, or flavour of the seaweed, but some participants did identify that seaweed could add texture to a product, which has also been identified in a past study by [62]. These responses also agree with a study by Senthil et al. [63] that incorporated seaweed into a spice mix as well as a study where brown seaweed was used to season corn snacks [64]. 

The participants were also asked to identify the flavour of seaweed. There was a great deal of agreement in the responses, as almost all participants identified salty or fishy or both. Both properties have been identified in sensory analysis trials on seaweed and seaweed-containing products [4,20,59,65,66]. This result also agrees with the participants’ responses to the first open-ended comment question, as participants thought seaweed could be used as “salt flavouring” (Seasoning-Table 2) and that the fishy taste of seaweed would work well with fish products (Fish-Table 2). Participants also stated that seaweed would taste earthy and bitter. Earthy or a mushroom aroma have been found to characterize red seaweeds [67], while bitterness has been associated with both red [66] and brown seaweed [68]. Many participants stated that the “earthiness is due to the bitterness of the seaweed.” It is interesting to note that the participants associated earthy and bitter to be very similar when discussing seaweeds. Some of the participants also indicated that the seaweed would taste savoury, as they felt seaweed could be added to savoury food items, which agrees with the responses above. Savoury or umami has also been identified in past studies as a taste associated with seaweed [68,69]. 

Lastly, participants were asked if they thought seaweeds had nutritional benefits, and overwhelmingly, the participants responded that they did. This result agrees with a past study on Australian consumers’ attitudes towards seaweeds [42] and indicates that consumers are aware that seaweed is a healthy option. Participants identified four general categories, including fibre, vitamins/nutrients, heart disease, and antioxidants. The participants were able to identify many different nutritional benefits that have been identified in past studies [52,70,71,72,73]. Other studies on consumer attitudes towards seaweeds have stated that education on the health benefits of seaweeds could increase consumption and acceptance of seaweed [74,75]. 

### 3.2. Segmentation

**Figure 2:**
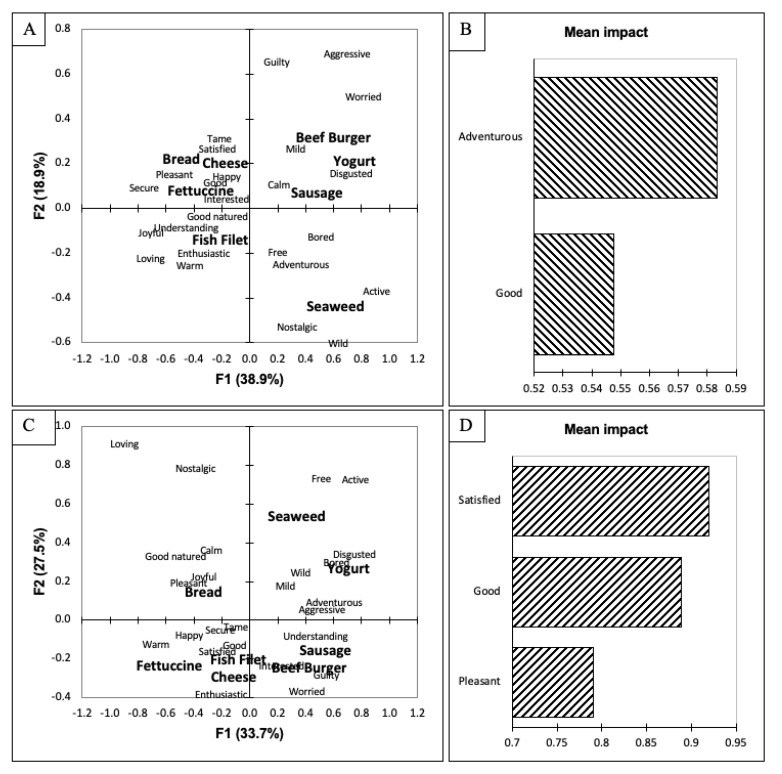
(**A**) Biplot representation of the seaweed-containing food items and emotional terms on the first two dimensions of the correspondence analysis for the population categorized as low hunger (LH). (**B**) Penalty lift analysis of the emotional terms and willingness to pay based on the LH population’s evaluation of the seaweed-containing food items. (**C**) Biplot representation of the seaweed-containing food items and emotional terms on the first two dimensions of the correspondence analysis for the population categorized as high hunger (HH). (**D**) Penalty lift analysis of the emotional terms and willingness to pay based on the HH population’s evaluation of the seaweed-containing food items.

The secondary objective was to evaluate how hunger status, food neophobia, and lifestyle affect consumers’ emotional responses. The participants were separated into different groups based on their responses to the respective scales. First, the participants were separated based on their hunger status into low hunger (LH, n = 54, sum score = 6.0–15.3) and high hunger (HH, n = 54, sum score = 15.9–29.7). The liking scores between the two groups were then compared using a *t*-test (Table 1). The only product that was significantly different between the two groups was the sausage, which the HH participants scored significantly lower than the LH group (*p* < 0.05). The CATA results of the two groups were then evaluated using CA and a penalty lift analysis (Figure 2). Both biplots (Figure 2A,C) were quite similar when looking at the first dimension separating the beef burger, yogurt, sausage, and seaweed from the bread, cheese, fettuccine, and fish filet. However, the LH (Figure 2A) separated the seaweed from the beef burger, yogurt, and sausage on the second dimension. Additionally, the seaweed was mainly associated with wild, nostalgic, active, adventurous, and free. The HH (Figure 2C) grouped the seaweed with the yogurt and associated it with wild, free, and active but also with bored and disgusted. The sausage was grouped with the beef burger and was associated with understanding, interested, guilty, and worried. The difference between the two groups becomes evident when looking at the penalty lift analyses (Figure 2B,D). The LH’s purchase-intent scores increased when adventurous and good were selected, while the HH’s purchase-intent scores increased when satisfied, good, and pleasant were selected. All of these emotions are categorized as positive emotions and have been shown to impact overall liking scores [49]. The LH participants’ purchase-intent scores were significantly impacted by the adventurous emotion. This result may indicate that since these participants are not hungry or satiated, they are willing to be more adventurous in their food choices. The HH purchase-intent scores increased when they selected the term satisfied. These participants may have been hungry at the time and wished to be satiated. When hungry, most humans want to be satiated [76], and this may have been seen in the results based on the impact of the emotional responses. Furthermore, de-Magistris and Gracia [77] found that hungry participants were more likely to pay a higher premium price for cheese than satiated participants. de-Magistris and Gracia [77] also found that participants overvalued the food product when they were hungry. This is the first study in which the authors are aware of what connects hunger status with emotional responses (EsSense25 Profile^®^; [27]). Furthermore, this study demonstrated that hunger status did impact the emotional responses chosen by participants as well as the purchase-intent scores. Future studies should be conducted to confirm these findings.

**Figure 3:**
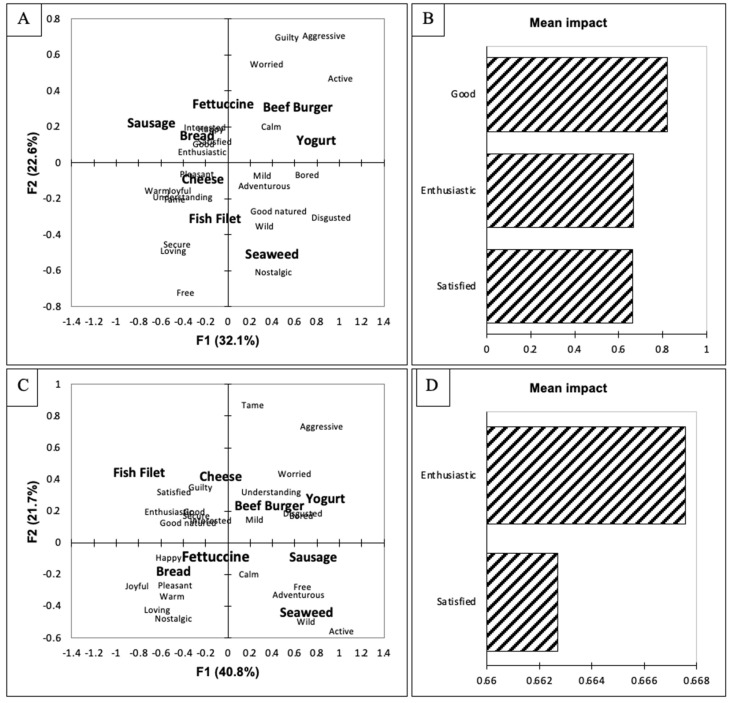
(**A**) Biplot representation of the seaweed-containing food items and emotional terms on the first two dimensions of the correspondence analysis for the population categorized as lower food neophobia (LFN). (**B**) Penalty lift analysis of the emotional terms and willingness to pay based on the LFN population’s evaluation of the seaweed-containing food items. (**C**) Biplot representation of the seaweed-containing food items and emotional terms on the first two dimensions of the correspondence analysis for the population categorized as higher food neophobia (HFN). (**D**) Penalty lift analysis of the emotional terms and willingness to pay based on the HFN population’s evaluation of the seaweed-containing food items.

The participants were also separated based on their responses to the FNS. They were separated into high food neophobia (HFN; n = 54, sum score = 30–48) and low food neophobia (LFN; n = 54, sum score = 12–29). There were no significant differences between the two groups for their purchase-intent scores (Table 1). However, for all food items except for the fish filet, the HFN group had lower purchase-intent scores. Food neophobia has been found to negatively impact consumer liking of food products [78]; however, as the participants did not consume the products in this study, the effect of food neophobia may have not been found. In both biplots (Figure 3A,C), the first dimension separated the sausage, beef burger, and yogurt from the fettuccine, bread, cheese, and fish filet. On the second dimension, the LFN separated the seaweed, cheese, and fish filet from the fettuccine, bread, sausage, beef burger, and yogurt. While the HFN group separated the bread and seaweed from the other food items, the biggest difference between the two groups was the placement of the bread. The LFN group associated the bread with happy, interested, satisfied, good, and enthusiastic, while the HFN group associated the bread with joyful, pleasant, warm, loving, and nostalgic. Overall, the bread was associated with positive emotions by both groups even though different positive emotions were identified. The penalty lift analyses (Figure 3B,D) demonstrated a difference between the two groups as well. The emotions of enthusiastic and satisfied increased the purchase intent of both groups; however, only the emotion of good increased the purchase-intent scores of the LFN group. This result may indicate that the HFN group did not associate many of the foods with good and would not purchase them. However, this thought is contradicted by the impact of the enthusiastic term used by the HFN group. This result may have occurred because the participants did not actually consume the food but rather just looked at pictures of food items. In a study in which consumers consumed sustainable food products, food neophobia did not affect consumers emotional responses [79]. Future studies need to be completed to investigate food neophobia and how it affects emotional responses to food items.

**Figure 4:**
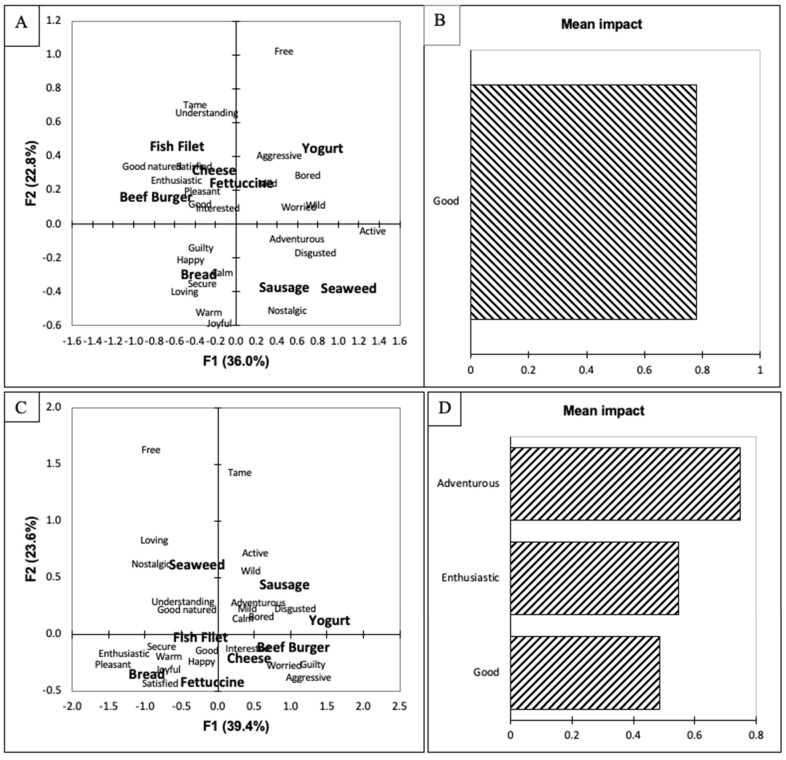
(**A**) Biplot representation of the seaweed-containing food items and emotions terms on the first two dimensions of the correspondence analysis for the population categorized as lower food-related lifestyle (LFRL). (**B**) Penalty lift analysis of the emotional terms and willingness to pay based on the LFRL population’s evaluation of the seaweed-containing food items. (**C**) Biplot representation of the seaweed-containing food items and emotional terms on the first two dimensions of the correspondence analysis for the population categorized as higher food-related lifestyle (HFRL). (**D**) Penalty lift analysis of the emotional terms and willingness to pay based on the HFRL population’s evaluation of the seaweed-containing food items.

Lastly, the participants were separated based on their responses to the FRL. There was one significant difference between the two groups for their purchase-intent scores (Table 2). The HFRL (higher food-related lifestyle, n = 54, sum score = 82–101) group liked the fish filet significantly more than the LFRL (lower food-related lifestyle, n = 54, sum score = 57–81) group (*p* < 0.05). In addition, the purchase-intent scores for the HFRL group were higher than the LFRL group except for the sausage, in which both groups scored it the same. The biggest difference between the groups’ emotional responses is evident in the biplots (Figure 4A,C), as bread was separated from the other items and was associated with happy, guilty, calm, secure, loving, warm, and joyful by the LFRL group. The HFRL associated the bread with the fettuccine and fish filet and associated the bread with the terms pleasant, joyful, warm, secure, happy, good, enthusiastic, and pleasant. On both biplots, sausage, yogurt, and seaweed were separated from the other food items (Figure 4A by the first dimension and Figure 4C by the second dimension). The penalty lift analysis indicates a clear difference between the two consumer groups. The LFRL group purchase-intent scores were positively impacted by the term good (Figure 4B), while the HFRL group’s scores were positively impacted by the terms adventurous, enthusiastic, and good (Figure 4D). The FRL attempts to capture the differences in how consumers view food and beverages [32]. Those that score highly on the FRL are considered to be foodies [80] and variety seekers [81]. The participants who scored highly on the FRL indicate that food is very important to them, and they spend considerable time and resources on food [32]. In addition, food has a major role in achieving their life values [32]. With food having this importance in their life, it may explain why adventurous and enthusiastic terms positively impacted their purchase-intent scores of the proposed seaweed products. The FRL identified differences in the participants’ emotional responses to seaweed-containing food items. More studies need to be completed to determine how FRL impacts the emotional responses of consumers. 

### 3.3. Limitations

This study presented an evaluation of how consumers perceive seaweed addition to food products, but some limitations need to be identified. Firstly, the emotional responses and purchase-intent scores were evaluated by looking at pictures of the food items. The results may have differed if participants consumed the food items. Secondly, the participants’ familiarity with consuming seaweed could have been evaluated, as familiarity has been found to influence participants’ liking and emotional responses to food products [82,83]. Additionally, the participants’ response to conventional food items (without seaweed) was not evaluated. Future studies should investigate how seaweed addition to food items changes consumers’ purchase intent. From a methodology perspective, the CATA questionnaire can be restrictive for participants in their choice of emotional responses. More studies are needed to confirm these results and to determine how hunger and FRL impact consumers’ emotional responses. In addition, more studies are needed to determine how emotional responses and purchase-intent scores are intertwined. Furthermore, the cultural background of the participants could have been evaluated, as it may have affected the FRL, food neophobia, emotional responses, and purchase-intent scores.

## 4. Conclusions

Purchase-intent scores were highest for bread and dried seaweed. The participants did not want to purchase yogurt and sausages with added seaweed. The dried seaweed and bread were associated with positive emotions and nostalgia, as outlined in the open-ended comment questions. Participants identified negative emotions when evaluating the beef burger, sausage, and yogurt, indicating that they were disgusted with the thought of seaweed being added to these food items. The purchase-intent scores increased when positive emotions (good, adventurous, and pleasant) were selected based on the penalty lift analysis. The participants identified that seaweed would work well with fish, savoury, and cereal grain-based food products as well as a seasoning or hidden in food products to promote nutritional benefits. Participants identified that seaweed has salty, fishy, earthy, bitter, and savoury flavours and associated it with many health benefits. The participants were also segmented based on their responses to hunger scales, FNS, and FRL. Hunger status as well as food neophobia and lifestyle all impacted participants emotional responses. Future research should expand on this study and evaluate how hunger and FRL impact emotional responses. In addition, future studies should investigate emotional responses to the consumption of seaweed-containing food products as well as investigate how familiarity and cultural background affect consumers’ emotional responses to new food products.

## Figures and Tables

**Table 1:** Consumer mean (±standard deviation) purchase-intent scores for the different food items.

	Overall Population (n = 108)	Low Hunger (LH)	High Hunger (HH)	Low Food Neophobia (LFN)	High Food Neophobia (HFN)	Lower Food-Related Lifestyle (LFRL)	Higher Food-Related Lifestyle (HFRL)
Bread	6.6a ± 1.2 ^1,2^	6.8a ± 1.0	6.4ab ± 0.9	6.7a ± 0.9	6.1ab ± 1.0	6.2a ± 0.8	6.7a ± 1.0
Dried Seaweed	6.4a ± 1.0	6.2ab ± 0.8	6.8a ± 1.1	6.7a ± 1.1	6.4ab ± 1.0	6.4a ± 0.9	6.9a ± 0.8
Fish Filet	6.0b ± 1.5	5.8ab ± 1.1	6.3ab ± 1.2	6.1ab ± 0.9	6.1abc ± 0.9	5.5ab ± 0.8 *	6.7a ± 0.9 *
Cheese	5.9bc ± 1.1	5.7ab ± 0.7	6.2ab ± 1.0	6.0ab ± 1.2	5.8abc ± 1.2	5.7ab ± 1.0	6.1ab ± 1.1
Beef Burger	5.9bc ± 1.3	5.7ab ± 0.7	6.2ab ± 1.1	6.0ab ± 1.1	5.8abc ± 1.1	5.7ab ± 1.1	6.1ab ± 1.0
Fettuccine [Noodles]	5.4bc ± 1.0	5.5ab ± 0.8	5.4bc ± 1.0	5.6abc ± 0.8	5.3abc ± 1.0	5.2ab ± 1.0	5.8bc ± 1.0
Yogurt	4.9c ± 1.3	5.1b ± 0.8	4.6cd ± 1.2	5.0bc ± 1.3	4.7bc ± 1.2	4.7b ± 1.0	5.1cd ± 1.1
Sausage [Pork]	4.5c ± 1.2	5.0b ± 1.2 *	3.9d ± 1.1 *	4.4c ± 1.0	4.7c ± 1.0	4.5b ± 1.3	4.5d ± 1.1

^1^ Means in the same column with the same letter are not significantly different at α = 0.05. ^2^ Data input on the 9-point purchase intent scale, where 1 = Definitely will not buy, and 9 = Definitely will buy. * Means between the population segments (LH vs. HH, LFN vs. HFN, LFRL vs. HFRL) differed based on a *t*-test.

**Table 2:** Results of the comment analysis conducted on the open-ended comments pertaining to seaweeds (n = 108).

Category	Summary of Responses and Keywords Identified
*If you had to use seaweed in a food product, what product would you add it to and why?*	
Fish	- Sushi, any fish products, fish [the taste might go well together], something with a fishy taste already, fish filets
Savoury	- Soup mix, savoury dish—such as sausage, potatoes, noodles, miso soup, eggs, ramen, cheese, casserole, easily disguised in savoury dishes, pesto sauce—herbs can mask the flavour
Hide it	- Meat sauce, casserole, smoothie—because I could hide the taste, salad dressing, pasta sauce to downplay the bitter taste, spaghetti sauce to mask the taste, add it to foods where it can be easily disguised
Cereal Grains	- Crackers, chips, bread, seaweed crackers, sourdough bread, pasta, grains-based products
Seasoning	- Salty flavouring to a variety of products, sprinkle on rice, compliment things with a strong taste, crunch factor, enhance flavour of ramen, add it to something that tastes mild to add a salty taste
*What do you think is the flavour of seaweeds and seaweed-containing products?*	
Salt	- Salt and the sea, brine taste, ocean, saltiness, a bit salty, salty, briny
Fishy	- Mild fishy, a little bit fishy, fishy but umami, fishy aftertaste,
Earthy	- Earthy, mushroom, grainy, grass-like, green, bitter—but earthy bitterness
Savoury	- Savoury, umami, mild savoury taste,
*Do you think seaweeds have any nutrition benefits, and if so, what are they?*	
Fibre	- Lots of fibre, roughage, healthy fibre
Vitamins/ Nutrients	- Iodine, minerals, iron, range of minerals, calcium, magnesium, potassium, omega-3, sodium, vitamin C, micronutrient, some vitamins, nutrient-dense
Heart Disease	- Reduces risk of heart disease, reduces major heart disease risks,
Antioxidants	- Antioxidants, phytochemicals, high in antioxidants, antioxidant characteristics

## Data Availability

The data presented in this study are available on request from the corresponding author (M.M).

## Supplementary Materials

The following are available online at https://www.mdpi.com/article/10.3390/foods10112737/s1, Table S1: Studies investigating incorporation of seaweed in different food products using sensory analysis, Table S2: Demographic details of the participants.
