# Supplementary material for: Do Consumers Want Seaweed in Their Food? A Study Evaluating Emotional Responses to Foods Containing Seaweed
Source: Foods. 2021 Nov 9;10(11):2737. doi: 10.3390/foods10112737 (PMC8621969; doi:10.3390/foods10112737)
Supplement: Supplementary file 1 [file foods-10-02737-s001.zip › foods-1434073-supplementary.pdf]

Supplementary Tables

Supplementary Table S1. Studies investigating incorporation of seaweed in different food products using sensory analysis.

Food item	Type of Panel	Reference
Fish filet	Trained panel	Deepitha et al., 2021
Pasta (durum semolina)	Semi-trained panel	Prabhasankar et al., 2009
Frankfurters	Trained panel	Jiménez-Colmenero et al., 2010
Chicken strips	Trained panel	Cofrades et al., 2011
Frankfurters	Consumer panel	Vilar et al., 2020
Iberico Cheese	Trained panel	del Olmo et al., 2018
Yogurt	Consumer panel	O'Sullivan et al., 2016
Salmon burgers	Trained panel	Dolea et al., 2018
Instant fried noodles	Consumer panel	Kumoro et al., 2016
Fish cutlet	Trained panel	Senthil et al., 2005
Beef burgers	Consumer panel	Cox & Abu-Ghannam, 2013
Yogurt and Quark	Trained panel	Nuñez & Picon, 2017
Cooked egg noodles	Trained panel	Chang & Wu, 2008
Fish jerky	Semi-trained panel	Hanjabam et al., 2017
Bread	Consumer panel	Lamont & McSweeney, 2021
Coffee beverage	Semi-trained panel	Kumar et al., 2019
Spice Mix	Trained panel	Senthil et al., 2011
Extruded Product	Trained panel	Singh et al., 2018
Muffin	Consumer Panel	Mamat et al., 2018

Supplementary Table S2. Demographic details of the participants.

Characteristics	**Participants (n=108)**
**Age**	
19-20	5
21-29	26
30-39	15
40-49	18
50-59	22
60-65	14
**Gender**	
Male	37
Female	63
Prefer not to say	0
**Income**	
Less than $25,000	10
$25,000-$44,999	20
$45,000-$64,999	14
$65,000-$99,999	22
$100,000-$149,000	6
$150,000+	6
Prefer not to say	22
**Education**	
Some High School	3
High School Graduate	18
Some Post-Secondary	20
Post-Secondary Certificate or Diploma	21
Bachelor's Degree	30
Above Bachelor's Degree	8
